# Prediction of Ultrasonic Pulse Velocity for Cement, Mortar, and Concrete through a Multiscale Homogenization Approach

**DOI:** 10.3390/ma15093241

**Published:** 2022-04-30

**Authors:** Jingluo Jiang, Dawei Zhang, Fuyuan Gong, Dian Zhi

**Affiliations:** College of Civil Engineering and Architecture, Zhejiang University, Hangzhou 310058, China; 21912049@zju.edu.cn (J.J.); dwzhang@zju.edu.cn (D.Z.); 22112046@zju.edu.cn (D.Z.)

**Keywords:** concrete, multiscale model, ultrasonic pulse velocity, homogenization approach, hydration process

## Abstract

Ultrasonic testing (UT) is an important method for concrete, and ultrasonic pulse velocity is commonly used to evaluate the quality of concrete materials in existing studies. The ultrasonic pulse velocity of concrete materials is affected by many factors; therefore, it is necessary to establish a quantitative prediction model for the ultrasonic pulse velocity of concrete materials. Based on the multiscale homogenization method, concrete material is divided into different scales of homogenized materials, namely cement paste, mortar, and concrete. Then, a multiscale ultrasonic pulse velocity model is established through a combination of elasticity formulation and the hydration model. At the three scales of cement paste, mortar, and concrete, the elastic parameters and ultrasonic pulse velocity were predicted with the water-to-cement ratio of 0.35, 0.5, and 0.65, respectively. The ultrasonic pulse velocity of concrete with different water-to-cement ratios and different ages were measured in the test and predicted by the model. The results show that the predicted value of ultrasonic pulse velocity is within the error range of ±1.5% of the measured ultrasonic pulse velocity, suggesting that the established prediction model of ultrasonic pulse velocity can reliably predict the velocity change in concrete materials.

## 1. Introduction

Ultrasonic testing (UT) is a commonly used concrete testing method, with advantages including fast testing speed, convenient operation procedure, and a non-destructive nature. The strength and other parameters of concrete can be evaluated based on its ultrasonic pulse velocity (UPV) [1,2,3]. It is critical to quantify the correlation of the required parameters with the ultrasonic pulse velocity of concrete. Some scholars have obtained empirical formulas by fitting a large amount of data, which are used to determine the strength of concrete relying on ultrasonic pulse velocity [4]. However, empirical correlation formulas cannot be applied to concrete of different conditions and properties. For special types of concrete, such as fiber reinforced concrete [5] and recycled aggregates concrete [6], the correlation between parameters needs to be re-given through experimental data analysis. In addition, many scholars have used ultrasonic pulse velocity to evaluate the damage of concrete [7], and have further completed the imaging of non-uniform damage [8,9]. Consequently, accurate ultrasonic pulse velocity models are needed to predict the UPV of the concrete for quantifying concrete performance. In some studies, the prediction model has been established to predict the strength [10] and porosity [11] of the concrete. Moreover, the forecasting progress can be used to investigate the development of the microstructure in the early hydration process [12].

In order to optimize the evaluation of concrete performance based on ultrasonic pulse velocity detection, it is necessary to study the ultrasonic pulse velocity prediction model of concrete materials. However, the ultrasonic pulse velocity of concrete is affected by various factors, such as the properties of materials [13], mix proportion [14], age, and moisture content [15]. Therefore, the accurate prediction of the ultrasonic pulse velocity of concrete materials remains an important research topic.

The mathematical model for predicting ultrasonic pulse velocity of concrete has been established by analysis of multiple influencing parameters [16]. Furthermore, with the model in the area of data analysis, a satisfactory prediction model can be obtained for ultrasonic pulse velocity [17]. In addition, in the existing studies, based on the elastic theory of homogeneous and isotropic materials, theoretical formulas for the correlation of elastic parameters and ultrasonic pulse velocity can be obtained through theoretical deduction [18]. In order to apply the elasticity formulation for homogeneous isotropic materials on non-homogeneous concrete, the homogenization approach of continuum micromechanics can provide a solution. In previous works, many scholars have studied the micromechanics model to obtain the uniform properties of the material from low-scale to high-scale, such as thermodynamic parameters [19,20], diffusion coefficient [21,22], and freezing behavior [23,24,25]. Many results in continuum micromechanics can be used for elastic parameter prediction of cement-based materials. Constantinides et al. [26] studied the elastic properties of two types of calcium silicate hydrates in cement-based materials through a micro-mechanical model and could predict the macro elasticity of cement paste with high accuracy. Ulm et al. [27] analyzed the applicability of the micromechanical model in concrete materials and studied the poroelasticity of multiscale and multiphase materials. Bernard et al. [28] successfully predicted the elastic modulus of concrete from the elastic parameters of microscopic hydrates by combining micromechanics and hydration models. Related studies on the prediction of elastic properties of cement-based materials through micromechanical models include Hellmich and Mang [29] and Pichler et al. [30]. Moreover, many scholars have studied the early elastic properties of cement-based materials, monitoring the hydration process by nonlinear elastic waves [31,32] and ultrasonic testing [33]. In this paper, the ultrasonic pulse velocity model of concrete materials was established by studying changes of elastic parameter in multiphase concrete materials.

In this study, based on the multiscale homogenization method and the elasticity formulation of homogenized multiphase material, a multiscale ultrasonic pulse velocity model for multiphase concrete material was established. Combined with the prediction of hydration process, the elastic modulus and ultrasonic pulse velocity could be predicted by the model in three-scale homogenized materials of cement paste, mortar, and concrete. The accuracy of the prediction model was verified by comparing the measured ultrasonic pulse velocity with the predicted value of the model. Based on this prediction model, the change of ultrasonic pulse velocity during the hydration process of concrete can be calibrated for future studies. Moreover, the concrete damage parameters can be introduced based on the established model for calculating the ultrasonic pulse velocity of concrete, which provides a theoretical basis for the study of ultrasonic pulse velocity of concrete damage.

## 2. Micromechanical Homogenization

Based on the elastic theory of homogeneous and isotropic materials, the formula for the correlation of elastic parameters such as ultrasonic pulse velocity and elastic modulus can be obtained through the derivation of the theoretical formula. The ultrasonic wavelength used in concrete ultrasonic testing is larger than the size of the discontinuities existing inside concrete. Therefore, the ultrasonic waves do not interact with these inhomogeneities, and concrete can reasonably be regarded as a homogeneous material [18]. The correlation theoretical formula can be applied in the ultrasonic testing of concrete:

(1) $$V=\sqrt{\frac{E}{\rho }\cdot \frac{1-\nu }{\left(1+\nu \right)\cdot \left(1-2\nu \right)}}=\sqrt{\frac{K+4/3G}{\rho }}$$

where V is the ultrasonic pulse velocity of the homogeneous material; E is the elastic modulus of the material; ρ is the density of the material; ν is the Poisson’s ratio of the material; and K and G are the bulk and shear modulus of the material, respectively.

However, concrete is a composite of different components, the overall properties of which cannot be directly determined when viewed as a homogeneous material. Therefore, we need to determine the *K*, *G,* and *ρ* of the concrete first.

The focus is then on how to predict *K*, *G* and *ρ*, or the effective properties of multiscale multiphase concrete composite (*K*^hom^, *G*^hom^, *ρ*^hom^). In this paper, the iterative homogenization method is used in concrete such a multiphase material to predict the parameters of *K* and *G* at different scales.

**Figure 1:**
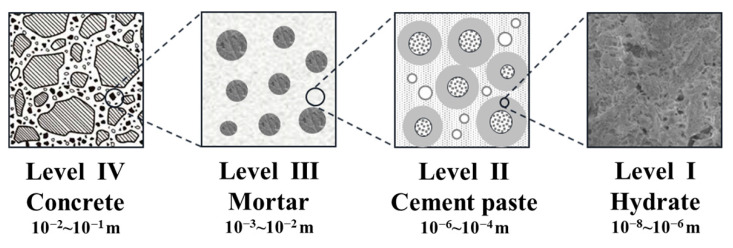
Multiscale homogenization scheme for concrete materials.

The homogenization method describes the overall elastic properties of representative volume elements (RVE) at different scales. The size of the representative volume unit defined within it needs to be much smaller than the overall analysis, and the characteristic length of the inhomogeneity in the unit needs to be much smaller than the size of the unit. In this scheme, the material in the RVE can be considered as uniform at the statistical level [34]. The elastic properties of higher-scale RVEs are described by the uniform structure at the micro scale. By repeatedly performing the homogenization scheme, the overall elastic properties of multiphase inhomogeneous materials can be predicted. For concrete, the multiscale division and homogenization scheme is shown in Figure 1.

According to the homogenization method, RVE selected at different scales can be regarded as uniform, so that the elastic parameters of cement-based materials at each scale are calculated according to the theory of micromechanics. Since each component is considered isotropic, the homogenized material is isotropic as well.

Combined with research results of micromechanics on the elasticity formulation of homogeneous multiphase materials, the authors in [35] summarize the following formulations for bulk modulus and shear modulus of homogeneous multiphase materials:

(2) $$\left\{ \begin{array}{l} K^{\mathrm{hom}}=\sum_{r=1}^{n}f_{r}K_{r}P_{r}/\sum_{r=1}^{n}f_{r}P_{r}\\ G^{\mathrm{hom}}=\sum_{r=1}^{n}f_{r}G_{r}Q_{r}/\sum_{r=1}^{n}f_{r}Q_{r}\\ \rho^{\mathrm{hom}}=\sum_{r=1}^{n}f_{r}\rho_{r} \end{array}\right.$$

where $K^{\mathrm{hom}}$, $G^{\mathrm{hom}}$, $\rho^{\mathrm{hom}}$, $K_{r}$, $G_{r}$, and $\rho_{r}$ are the bulk modules, shear modulus, density of homogenized material, and phase *r*, respectively; $P_{r}$, $Q_{r}$, and $f_{r}$ are the compressibility, shear compliance, and volume fraction of each phase *r,* respectively; and n is the number of phases in the composite.

The elastic modulus and Poisson’s ratio can be calculated according to the following Equation (3):

(3) $$\left\{ \begin{array}{l} E^{\mathrm{hom}}=\frac{9K^{\mathrm{hom}}G^{\mathrm{hom}}}{3K^{\mathrm{hom}}+G^{\mathrm{hom}}}\\ v^{\mathrm{hom}}=\frac{3K^{\mathrm{hom}}-2G^{\mathrm{hom}}}{6K^{\mathrm{hom}}+2G^{\mathrm{hom}}} \end{array}\right.$$

where $E^{\mathrm{hom}}$ and $\nu^{\mathrm{hom}}$ are the elastic modulus and Poisson’s ratio of homogenized material.

The compressibility *P* and shear compliance *Q* are determined by the shape and elastic properties of each phase in the material. In this study, it is considered that each component exists in a nearly spherical state in undamaged concrete. According to Berryman [36], the compressibility *P* and shear compliance *Q* are calculated considering spherical inclusions, as seen in Equation (4):

(4) $$\left\{ \begin{array}{l} P_{r}^{sph}=\frac{K_{0}+4G_{0}/3}{K_{r}+4G_{0}/3}\\ Q_{r}^{sph}=\frac{G_{0}+F_{0}}{G_{r}+F_{0}} \end{array}\right.$$

where $K_{0}$ and $G_{0}$ are the compressibility and shear compliance of the matrix phase, which represents the lower scale medium. The $F_{0}$ can be calculated as:

(5) $$F_{0}=\left(G_{0}/6\right)\left[\left(9K_{0}+8G_{0}\right)/\left(K_{0}+2G_{0}\right)\right]$$

According to Equations (2)–(5), the compressibility and shear compliance of homogeneous media at different scales can be obtained, depending on a suitable iteration scheme.

Three scales are considered in concrete materials: (1) cement paste composed of CSH skeleton and pore substances; (2) mortar composed of cement paste and fine aggregates; and (3) concrete composed of mortar and coarse aggregates. The Mori-Tanaka scheme [37] and self-consistent scheme [38] were selected for iterative calculation of the homogenization process of media at different scales. These two approaches are commonly used to predict the overall elastic properties of homogeneous and isotropic composite materials from the micromechanics point of view. There are some connections between them; however, different homogenization processes need corresponding schemes to evaluate the effective properties of composites.

Usually, the Mori-Tanaka scheme defines a medium as reference medium, which has a large volume fraction in the mixture. The self-consistent scheme takes each medium as the reference medium for step-by-step iterative calculation, which is suitable for a closer volume fraction of each phase. Significantly, it leads to different values being substituted in Equation (4) when different schemes are used. $K_{0}$ and $G_{0}$ are equal to $K^{\mathrm{hom}}$ and $G^{\mathrm{hom}}$ of the RVE at the lower scale when using the Mori-Tanaka scheme. In the self-consistent scheme, $K_{0}$ and $G_{0}$ refer to $K^{\mathrm{hom}}$ and $G^{\mathrm{hom}}$ of the overall medium at the previous iteration, which has multiple iterations at the same scale.

Thus, the self-consistent scheme is used for cement paste due to its complex components, including more overlaps among the components and more pores. Obviously, using the Mori-Tanaka scheme for mortar is more suitable because the volume fraction of cement paste is so large that it can be considered as the main body, and the fine aggregate as spherical inclusions. In the same way, the mortar can be considered as the main body and the coarse aggregate as spherical inclusions in the concrete level. We chose the Mori-Tanaka scheme for concrete. The prediction of elastic parameter and ultrasonic pulse velocity in this paper refers to the above selection of the iterative scheme.

## 3. Ultrasonic Pulse Velocity Model for Cement Paste

### 3.1. Hydration Model and Volume Fraction

The RVE of cement paste consists of unhydrated cement clinker, water, hydrates, and air voids [28,29,30,39]. From the hydration degree of 0 (initial) to the maximum (completed), the volume fraction of each material constantly evolves. Following the Powers-Acker model [40], the volume fraction of clinker (clin), water (H_2_O), hydrates (hyd), and air voids as functions of the hydration degree *ξ* and the water-to-cement ratio *w/c* are:

(6) $$f_{\mathrm{clin}}\left(\xi \right)=\frac{1-\xi }{1+\frac{\rho_{\mathrm{clin}}}{\rho_{H_{2}O}}\left(w/c\right)}=\frac{20\left(1-\xi \right)}{20+63\left(w/c\right)}\geq 0$$

(7) $$f_{H_{2}O}\left(\xi \right)=\frac{\rho_{\mathrm{clin}}\left[\left(w/c\right)-0.42\xi \right]}{\rho_{H_{2}O}\left[1+\frac{\rho_{\mathrm{clin}}}{\rho_{H_{2}O}}\left(w/c\right)\right]}=\frac{63\left[\left(w/c\right)-0.42\xi \right]}{20+63\left(w/c\right)}\geq 0$$

(8) $$f_{\mathrm{hyd}}\left(\xi \right)=\frac{1.42\rho_{\mathrm{clin}}\xi }{\rho_{\mathrm{hyd}}\left[1+\frac{\rho_{\mathrm{clin}}}{\rho_{H_{2}O}}\left(w/c\right)\right]}=\frac{43.15\xi }{20+63\left(w/c\right)}$$

(9) $$f_{\mathrm{air}}\left(\xi \right)=\frac{\left(1+0.42\frac{\rho_{\mathrm{clin}}}{\rho_{H_{2}O}}-1.42\frac{\rho_{\mathrm{clin}}}{\rho_{\mathrm{hyd}}}\right)\xi }{1+\frac{\rho_{\mathrm{clin}}}{\rho_{H_{2}O}}\left(w/c\right)}=\frac{3.31\xi }{20+63\left(w/c\right)}$$

where *ξ* is the hydration degree; *w/c* is the water-to-cement ratio; $f_{\mathrm{clin}}$, $f_{H_{2}O}$, $f_{\mathrm{hyd}}$, and $f_{\mathrm{air}}$ refer to the volume fraction of unhydrated cement clinker, water, hydrates, and air voids, respectively; and $\rho_{\mathrm{clin}}$, $\rho_{H_{2}O}$, and $\rho_{\mathrm{hyd}}$ refer to the mass densities of clinker, water, and hydrates, respectively, referencing the parameters in Table 1.

The maximum hydration degree is affected by the water-to-cement ratio. If the water-to-cement ratio is below 0.42, the cement clinker cannot be fully hydrated. The hydration degree reaches the maximum when the water is exhausted. If the water-to-cement ratio is larger than 0.42, the maximum hydration degree is equal to 1 when the cement clinker is fully consumed [30,39]. The maximum hydration degree follows as:

(10) $$\max\xi =\left\{ \begin{array}{cccc} \frac{\left(w/c\right)}{0.42} & \mathrm{for} & \left(w/c\right)\leq 0.42\Leftrightarrow f_{\mathrm{clin}}\left(\max\xi \right)\geq 0, & f_{H_{2}O}\left(\max\xi \right)=0,\\ 1 & \mathrm{for} & \left(w/c\right)\geq 0.42\Leftrightarrow f_{\mathrm{clin}}\left(\max\xi \right)=0, & f_{H_{2}O}\left(\max\xi \right)\geq 0. \end{array}\right.$$

where *ξ* is the hydration degree and *w/c* is the water-to-cement ratio. The hydration degree evolves with age, influenced by the water-to-cement ratio, aggregate properties, and so on. In order to evaluate the proportion of each component in the composites, we need a scheme to predict the hydration degree.

**Figure 2:**
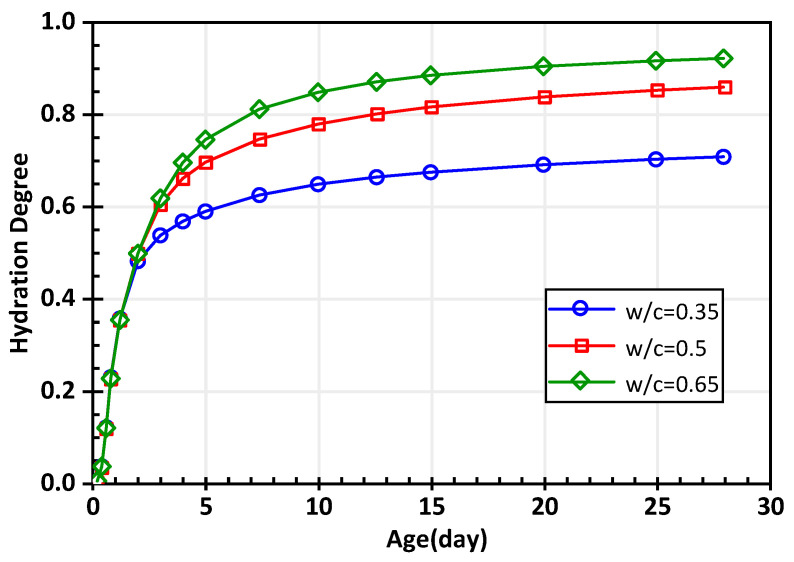
The predicted curve of the hydration degree increasing with age from the DuCOM model.

With the calculation of pore distribution and hydration heat by the DuCOM model [42], the increase of the hydration degree *ξ* with a specific water-to-cement ratio can be quantified. Different hydration processes with water-to-cement ratios of 0.35, 0.5, and 0.65 are shown in Figure 2.

Obviously, the hydration degree shows a notable acceleration since initiation, and a slight increase in the hydration degree was found in the stable period. Based on the analysis of the predicted results, the mixture with a larger water-to-cement ratio has a higher hydration degree at the same age before the process is ended.

### 3.2. Velocity Prediction

At the scale of cement paste (Level II), the prediction of ultrasonic pulse velocity requires correlation parameters of clinker, water, hydrates, and air voids to predict the elastic parameter, as shown in Table 1.

**Figure 3:**
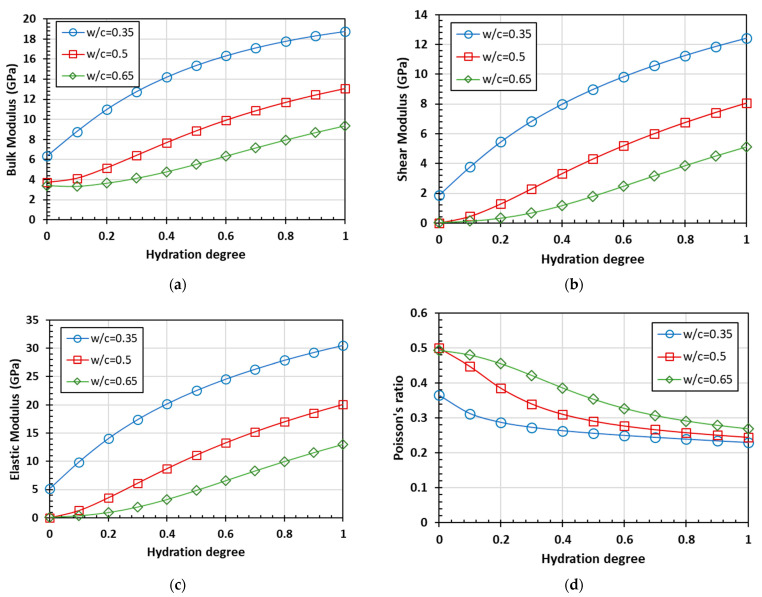
The prediction of elastic parameters based on the homogenization process: (**a**) bulk modulus; (**b**) sheer modulus; (**c**) elastic modulus; and (**d**) Poisson’s ratio.

According to Equations (2)–(5), bulk modulus *K*, shear modulus *G*, elastic modulus *E*, and Poisson’s ratio *υ* of homogenized material and phase were calculated by the model with the self-consistent scheme. The correlation curves between different parameters and the hydration degree are shown in Figure 3.

At the same degree of hydration, a higher water-to-cement ratio shows a lower bulk modulus, shear modulus, and elastic modulus. With the growing hydration degree, the bulk modulus, shear modulus, and elastic modulus of the cement paste all show an increasing trend, and the differences between these parameters also increase gradually. For cement paste with a high water-to-cement ratio in the initial stage of hydration, the shear modulus and elastic modulus increase from around 0, which is also close to the corresponding parameters of water. Poisson’s ratio decreases with the increase of the hydration degree, and Poisson’s ratios of cement paste with different water-to-cement ratios approach 0.25 when the hydration is completed.

**Figure 4:**
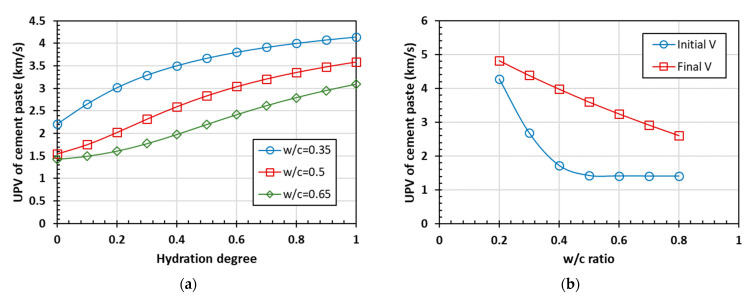
The ultrasonic pulse velocity prediction of cement paste: (**a**) hydration process and (**b**) the influence of the water-to-cement ratio.

The ultrasonic pulse velocity (UPV) of the cement paste can be calculated using Equation (1) with the elastic parameters. The UPV of cement paste as a function of the hydration degree is shown in Figure 4.

The predicted ultrasonic pulse velocity of the cement paste also increases with the degree of hydration, displaying the same trend as the bulk modulus, shear modulus, and elastic modulus. In the case of a high water-to-cement ratio, the initial value of the wave speed is about 1.5 km/s, which is close to the ultrasonic speed in water. When the hydration is completed, the influence of the water-to-cement ratio on the final UPV is generally linear with a negative correlation. The influence on the initial UPV is more sensitive when the water-to-cement ratio is less than 0.4. Once the water-to-cement ratio is greater than 0.4, its increase has little effect on the initial UPV. The main influencing factor on the final UPV is the proportion of hydrates in the cement paste. When the volume fraction of water, which mainly affects the initial UPV, is larger in the mixture, the ultrasonic pulse velocity will continue to approach the ultrasonic pulse velocity in water.

**Figure 5:**
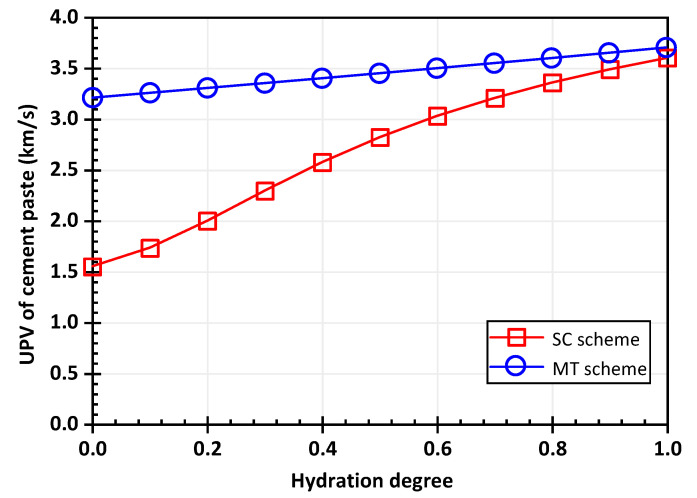
The ultrasonic pulse velocity prediction based on the different schemes.

Considering different schemes, the predicted results are shown in Figure 5. The ultrasonic pulse velocity obtained by the Mori-Tanaka scheme increases linearly with an increasing degree of hydration, starting from about 3.2 km/s. The velocity calculated by the self-consistent scheme gradually increases from about 1.5 km/s, and this value is close to the value of the ultrasonic pulse velocity in water. The cement paste includes hydration products, water, air voids, and unhydrated clinker. Before the hydration starts, the theoretical value of the wave speed is supposed to be close to the wave speed in water when the water-to-cement ratio is relatively large. Meanwhile, the internal components of the cement paste are complex, with impurities and multitudes of and pores, meaning that there is no component with a large volume ratio in the early stage of hydration. Therefore, the self-consistent scheme is adopted for calculation.

## 4. Ultrasonic Pulse Velocity Model Upgraded to Mortar and Concrete

### 4.1. Velocity Model for Mortar

When the ultrasonic pulse velocity model upgrades to mortar and concrete, the volume fraction of the aggregate in each scale material should be determined. According to the mix proportion and density of the aggregate, the volume fraction can be calculated using Equations (11) and (12).

(11) $$\left\{ \begin{array}{l} f_{sand\;}=\frac{M_{sand\;}/\rho_{sand\;}}{1-M_{grav\;}/\rho_{grav\;}}\\ f_{cem\;}=1-f_{sand\;} \end{array}\right.$$

(12) $$\left\{ \begin{array}{l} f_{grav}=\frac{M_{grav}}{\rho_{grav}}\\ f_{mot\;}=1-f_{grav\;} \end{array}\right.$$

where $f_{sand\;}$ and $f_{cem\;}$ are the volume fraction of fine aggregate and cement paste in the mortar, respectively; $f_{grav}$ and $f_{mot\;}$ are the volume fraction of coarse aggregate and mortar in the concrete, respectively; $M_{sand\;}$ and $M_{grav\;}$ are the amount of the fine aggregate and coarse aggregate in mix proportion, respectively; and $\rho_{sand\;}$ and $\rho_{grav\;}$ are the density of the fine and coarse aggregate, respectively.

The quantified prediction of the mortar and concrete also requires the elastic parameters of the cement paste, while the required physical parameters of the aggregate are shown in Table 2.

The parameter can be given for the prediction of ultrasonic velocity, which is calculated according to the commonly used mix proportion, as shown in Table 3.

**Figure 6:**
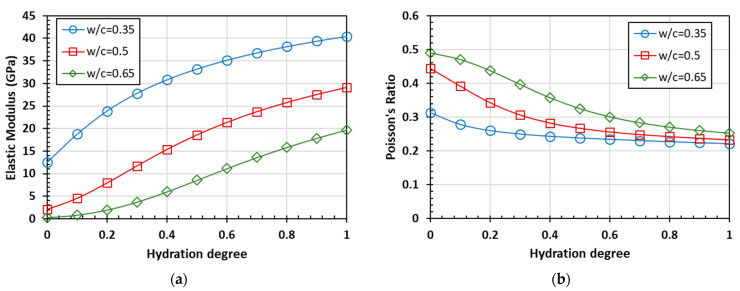
The predicted elastic parameters of mortar as a function of hydration degree: (**a**) elastic modulus and (**b**) Poisson’s ratio.

**Figure 7:**
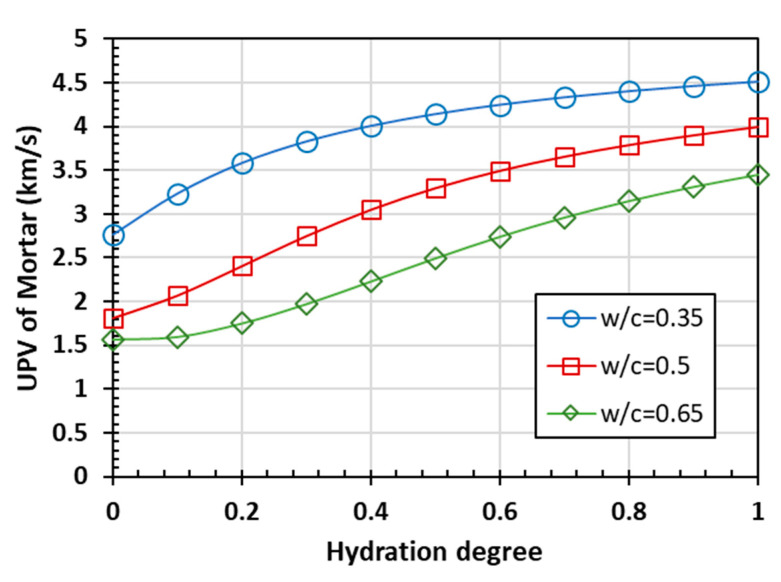
The ultrasonic pulse velocity prediction of mortar.

The calculated results indicate that the whole cement paste is taken as one phase in the two-phase material composed of cement paste and fine aggregate. According to the established prediction model, the curves of the elastic modulus and Poisson’s ratio of mortar are predicted with the Mori-Tanaka scheme, as shown in Figure 6. Then, the predicted UPVs of mortar with different *w/c* ratios, which are shown in Figure 7, can be easily obtained using Equation (1).

The overall trend of the predicted curve is consistent with the predicted results of the cement paste. Compared with cement paste, the elastic modulus and ultrasonic pulse velocity of mortar are larger, while the Poisson’s ratio is smaller at the same hydration degree.

### 4.2. Velocity Model for Concrete

According to the preceding homogenization process of mortar scales, the mortar is considered one phase to the composite, considering the concrete as a two-phase material consisting of mortar and coarse aggregate. With the calculating parameters of the concrete scale shown in Table 2 and Table 3, the predicted results can be obtained by the preceding calculated process with the Mori-Tanaka scheme.

**Figure 8:**
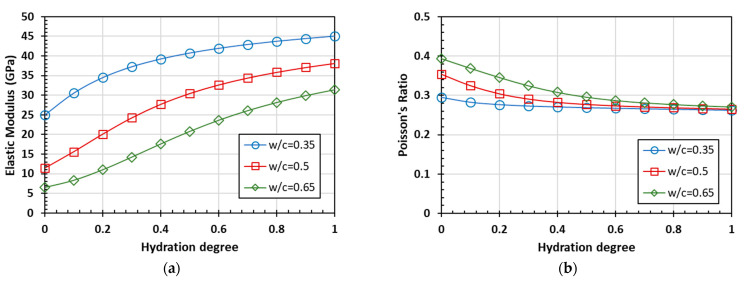
The predicted elastic parameters of concrete as functions of the hydration degree: (**a**) elastic modulus and (**b**) Poisson’s ratio.

**Figure 9:**
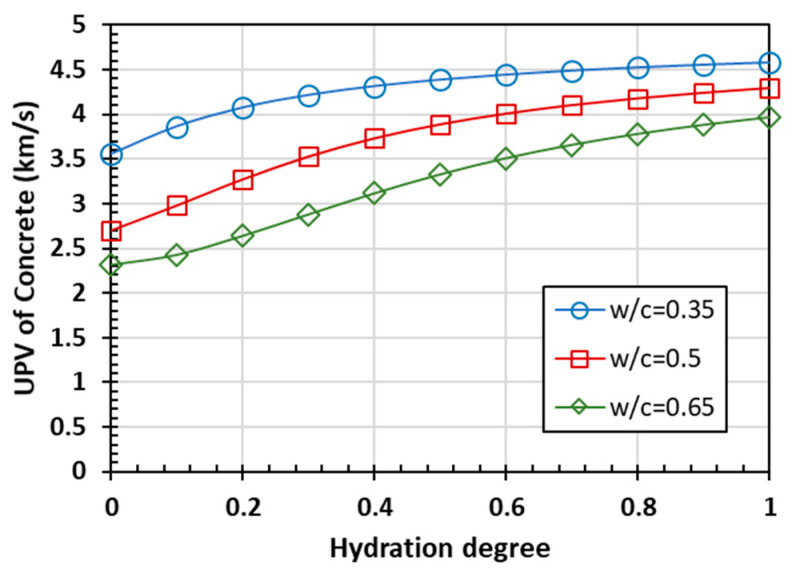
The ultrasonic pulse velocity prediction of concrete.

The predicted results for the elastic modulus, Poisson’s ratio, and UPV of concrete as functions of the hydration degree are shown in Figure 8 and Figure 9.

The overall trend of the predicted curves is consistent on the three scales of cement paste, mortar, and concrete. However, the aggregate, occupying the bulk of the volume, has stable properties and basically unchanged relevant parameters with the hydration process. This means that with the progress of hydration, the differences between the prediction values of the elastic modulus, Poisson’s ratio, and ultrasonic pulse velocity corresponding to different water-to-cement ratios are decreasing. Due to the addition of the aggregate phase, the ultrasonic pulse velocity of the homogenized materials at the scale of mortar and concrete increases at the same degree of hydration, compared with the scale of cement paste. In the case of the same water-to-cement ratio from the initial stage of hydration to completed hydration, the change of ultrasonic pulse velocity is also smaller than that of the cement paste scale.

## 5. Experimental Verification

### 5.1. Experimental Setup

To verify the accuracy of the multiscale ultrasonic pulse velocity prediction model, a validation experiment was designed.

Three series of concrete specimens were prepared with two different target ages (7 and 28 days) and three different *w/c* ratios (0.37, 0.42, and 0.55). Ordinary Portland cement with a grade of P.O 32.5 was used. The fine aggregate used for the concrete was local river sand with a fineness modulus of 2.3 and a relative density of 2.65. The coarse aggregate used for concrete was local limestone, with sizes of 5–20 mm and a relative density of 2.62. The hydration degree of the three concrete mixtures at the ages of 7 days and 28 days were predicted by the DuCOM model, which is shown in Table 4, as well as the volume fraction of components at each age.

The concrete specimens with dimensions of 100 mm × 100 mm × 100 mm were demolded after 48 h. Each sample was placed in water to cure at curing room temperature (i.e., 20 ± 2 °C) for 7 days or 28 days.

**Figure 10:**
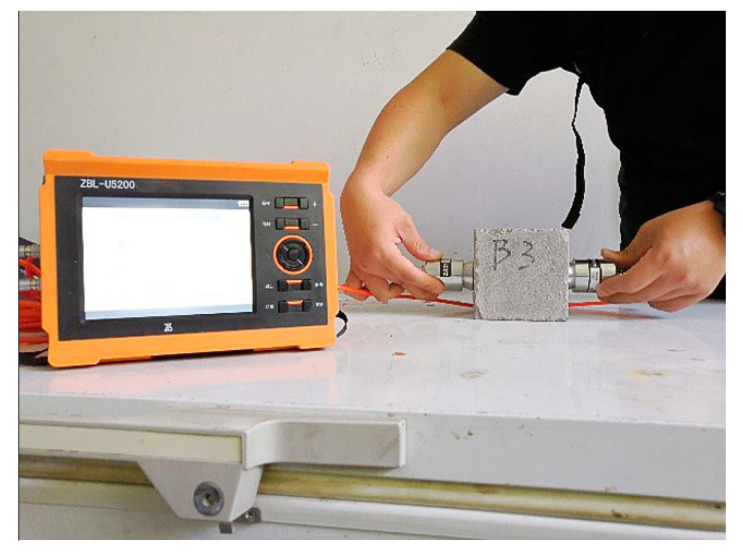
The measurement of ultrasonic pulse velocity of concrete specimen.

The specimens and UPV testing tools are shown in Figure 10; the UPV of the specimen from three concrete types were measured at the age of 7 days and 28 days.

**Figure 11:**
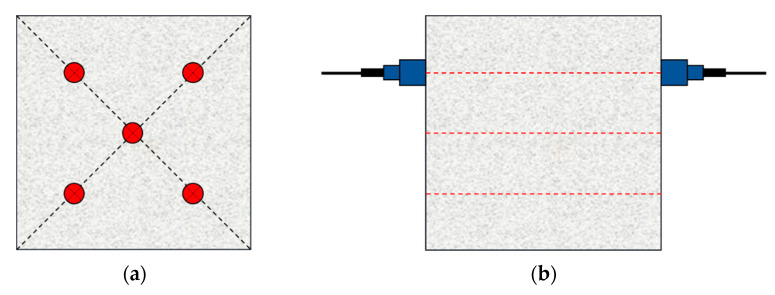
The measurement of ultrasonic pulse velocity: (**a**) layout of measuring points and (**b**) direct transmission.

The “ZBL-U5200” device was adopted to measure the UPV of the concrete specimen. The measurement of UPV was carried out immediately after the specimen left the curing condition to ensure the stability of its moisture content and other states. The transducers were placed on two parallel surfaces of the specimen, with the Vaseline coupling between the transducers and the specimen. Five measuring points were designed on two parallel surfaces, with three instances of measurements on each measuring point. The ultrasonic pulse velocity detection test adopts the direct transmission, as shown in Figure 11.

### 5.2. Experimental Results and Comparison

**Figure 12:**
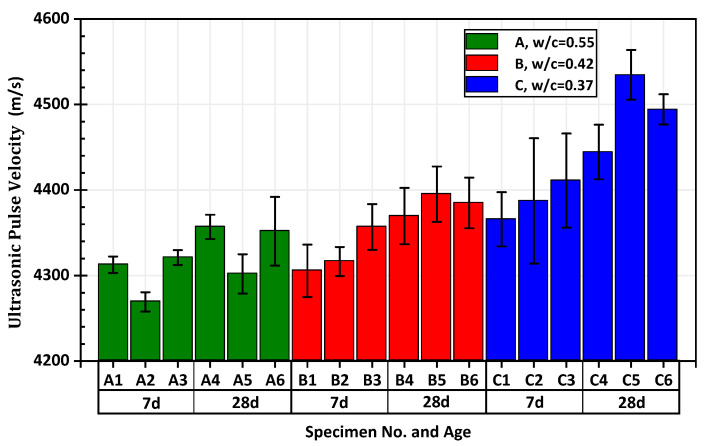
The result of ultrasonic testing with standard error.

The testing results of the UPV are shown in Figure 12.

With the increase of age, the ultrasonic pulse velocities of groups A, B, and C showed an overall increasing trend. At the same age, the ultrasonic pulse velocity of the specimens in the low water-to-cement ratio group was significantly higher than that in the high water-to-cement ratio group. The error bar shows that the standard error of the measured value of each specimen in the test was also controlled within a reasonable range.

**Figure 13:**
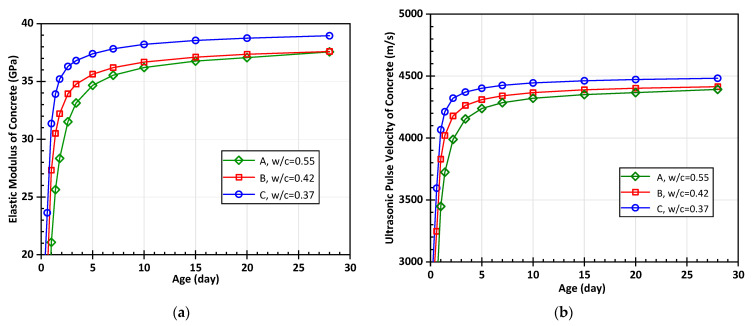
The predicted curves at different ages based on the multiscale ultrasonic pulse velocity prediction model: (**a**) elastic modulus and (**b**) ultrasonic pulse velocity.

According to the experimental material parameters, the elastic modulus and UPV of concrete can be predicted based on the multiscale ultrasonic pulse velocity prediction model, as shown in Figure 13.

It can be observed that the trend of ultrasonic pulse velocity is consistent with that of the hydration degree. In the early stage of hydration, due to severe hydration reaction, the ultrasonic pulse velocity and elastic modulus increase rapidly, reaching about 80% of the maximum value within 5 days of age. The development trend then slows down. At 28 days of age, the speed is extremely slow or even remains unchanged.

For concrete at the same age, the ultrasonic velocity of those with a water-to-cement ratio of 0.55 is the smallest, which develops to about 4300 m/s at 28 d. The ultrasonic velocity of concrete with a water-to-cement ratio of 0.37 is the highest, which finally develops to about 4500 m/s. Although concrete with a high water-to-cement ratio has a higher degree of hydration at the same age, its elastic model and ultrasonic pulse velocity values are still lower than those of low water-to-cement ratio concrete. Therefore, the mix design has a great influence on the elastic parameters and ultrasonic pulse velocity of the concrete material.

**Figure 14:**
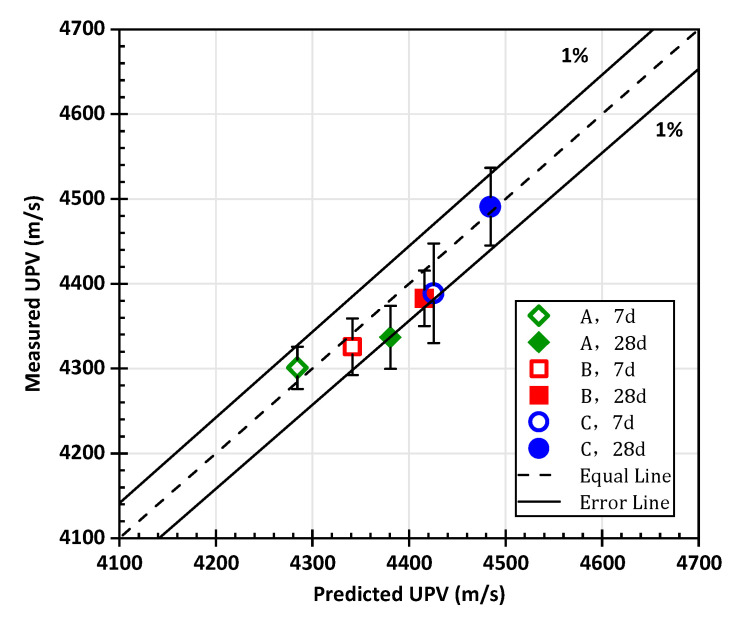
The comparison diagram between the predicted value of the multiscale UPV model and the measured value.

Comparing the predicted results with the measured results, as shown in Figure 14, it can be found that the result points are close to the equal line. The numerical simulation is in good agreement with the experiment, indicating the accuracy of the multiscale ultrasonic pulse velocity model.

The standard deviation of the measured values of each group is marked in Figure 14, showing that the dispersion of the experiment results remains within a reasonable range. The error of the predicted UPV relative to the experimental measured UPV is basically within ±1%. In the group of 28 days age, the relative error with a water-to-cement ratio of 0.55 is slightly larger, but is still within the range of ±1.5%.

The predicted ultrasonic pulse velocity values highly fit with the measured values, showing a satisfactory agreement. If the required parameters are available, the elastic parameters and UPV of the multiphase cement-based material can be predicted by this multiscale ultrasonic pulse velocity model.

With more experimental data, further research will be able to verify the applicability of this new calculation model in other cases.

**Figure 15:**
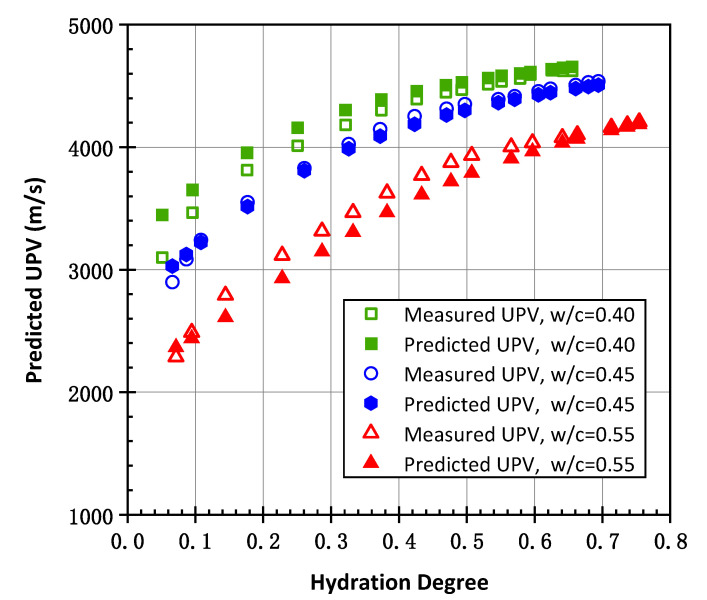
The comparison between predicted UPV and measured UPV of the hydration process with different *w/c* ratios.

According to Ye’s study [12], the ultrasonic pulse velocity of concrete was calculated by the multiscale theoretical calculation model in this paper with the experimental parameters. Figure 15 shows the results obtained by the theoretical model with different *w/c* ratios in Ye’s study, compared with the measured results.

The results indicate that the predicted UPV of concrete is close to the measured UPV, especially after reaching a high hydration degree. It is thought that the air entrapped in the fresh cement paste is the reason for random error in the initial stage, which is more pronounced in the case of high *w/c* ratios. In addition, the effect of the different cement materials’ properties provided in the paper also leads to some random errors. In general, the multiscale ultrasonic pulse velocity model exhibits a good predictive capacity when it is adapted to predict the ultrasonic pulse velocity of concrete.

## 6. Conclusions

In this paper, a multiscale ultrasonic pulse velocity model for multiphase concrete materials is established based on the homogenization approach combined with the model of the hydration process. During the hydration process, the elastic parameters and ultrasonic pulse velocity of cement paste, mortar, and concrete can be predicted by the model. The accuracy of this model is verified by concrete ultrasonic testing and other research data.

1. Based on the multiscale homogenization method and the elasticity formulation of homogenized multiphase materials, a multiscale ultrasonic pulse velocity model is established which can predict elastic parameters and ultrasonic pulse velocity during the hydration process, according to material parameters, mixture, and age.
2. In this model, the iterative calculation of elastic parameters requires different schemes at different scales. The self-consistent scheme is applied at the scale of cement paste, while the Mori-Tanaka scheme is used at the mortar and concrete scales.
3. The elastic parameters and ultrasonic pulse velocity at three scales of cement paste, mortar, and concrete were predicted by this model with the water-to-cement ratios of 0.35, 0.5, and 0.65, respectively. At the scale of cement paste, the volume fraction of water and the proportion of hydrates are the main influencing factors of the initial ultrasonic pulse velocity and final ultrasonic pulse velocity, respectively. At the scale of mortar and concrete, the aggregates make the influence of the water-to-cement ratio gradually decrease as hydration progresses, with the large volume fraction and stable nature.
4. According to the experiments detailed in this paper, the relative error of the measured ultrasonic pulse velocity and the predicted value is within ±1.5%. According to Ye’s study [12], the ultrasonic pulse velocity of concrete was predicted by the prediction model, and the obtained prediction value agreed well with the measured value. Thus, the model is reliable for predicting the ultrasonic pulse velocity of concrete materials. This has reference value for the prediction of cement-based ultrasonic pulse velocity. However, for special concrete, the elastic parameters of special aggregates are very different from that of ordinary concrete, which cannot be ignored in the prediction of elastic parameters. Research will be carried out on the prediction of ultrasonic pulse velocity for special concrete in the future.

## Figures and Tables

**Table 1:** Physical parameters of different phases for cement paste.

Phase	Bulk Modulus *K* (GPa)	Shear Modulus *G* (GPa)	$\mathrm{Density}\rho \;({\mathrm{kg/m}}_{3})$	References
Cement (clinker)	116.7	53.8	3150	[29,30,39]
Water	2.3	0	1000	[29,30,39]
Hydrates	18.7	11.8	2073	[26,27,28,41]
Air	0	0	0	[29,30,39]

**Table 2:** Physical parameters of the aggregate.

Phase	Bulk Modulus *K (*GPa)	Shear Modulus *G (*GPa)	$\mathrm{Density}\rho \;({\mathrm{kg/m}}_{3})$	References
Fine Aggregate	36	26	2650	[27]
Coarse Aggregate	41.6	19.2	2620	[29,39]

**Table 3:** The parameters for prediction.

*w/c* Ratio	Volume Fraction of Fine Aggregate in Mortar	Volume Fraction of Coarse Aggregate in Concrete
0.35	0.404	0.5
0.5	0.358	0.5
0.65	0.311	0.5

**Table 4:** The parameters of experimental materials.

Group	Age (Days)	*w/c* Ratio	Hydration Degree	Volume Fraction of Fine Aggregate in Mortar	Volume Fraction of Coarse Aggregate in Concrete
A	7	0.55	0.760	0.491	0.482
	28	0.55	0.880	0.491	0.482
B	7	0.42	0.676	0.350	0.420
	28	0.42	0.780	0.350	0.420
C	7	0.37	0.620	0.319	0.420
	28	0.37	0.710	0.319	0.420

## Data Availability

Data is contained within the article. The data presented in this study are available in [12] and this paper.
